# An adaptable chromosome preparation methodology for use in invertebrate research organisms

**DOI:** 10.1186/s12915-018-0497-4

**Published:** 2018-02-26

**Authors:** Longhua Guo, Alice Accorsi, Shuonan He, Carlos Guerrero-Hernández, Shamilene Sivagnanam, Sean McKinney, Matthew Gibson, Alejandro Sánchez Alvarado

**Affiliations:** 1 University of California, Los Angeles, CA USA; 2 Stowers Institute for Medical Research, Kansas City, MO USA; 3 Howard Hughes Medical Institute, Kansas City, MO USA; 4 Oregon Health and Science University, Portland, OR USA

**Keywords:** Colchicine, Karyotype, Karyogram, Planarian, *Schmidtea mediterranea*, *Dugesia japonica*, Gastropod, Apple snail, *Ampullariidae*, *Nematostella*

## Abstract

**Background:**

The ability to efficiently visualize and manipulate chromosomes is fundamental to understanding the genome architecture of organisms. Conventional chromosome preparation protocols developed for mammalian cells and those relying on species-specific conditions are not suitable for many invertebrates. Hence, a simple and inexpensive chromosome preparation protocol, adaptable to multiple invertebrate species, is needed.

**Results:**

We optimized a chromosome preparation protocol and applied it to several planarian species (phylum Platyhelminthes), the freshwater apple snail *Pomacea canaliculata* (phylum Mollusca), and the starlet sea anemone *Nematostella vectensis* (phylum Cnidaria). We demonstrated that both mitotically active adult tissues and embryos can be used as sources of metaphase chromosomes, expanding the potential use of this technique to invertebrates lacking cell lines and/or with limited access to the complete life cycle. Simple hypotonic treatment with deionized water was sufficient for karyotyping; growing cells in culture was not necessary. The obtained karyotypes allowed the identification of differences in ploidy and chromosome architecture among otherwise morphologically indistinguishable organisms, as in the case of a mixed population of planarians collected in the wild. Furthermore, we showed that in all tested organisms representing three different phyla this protocol could be effectively coupled with downstream applications, such as chromosome fluorescent in situ hybridization.

**Conclusions:**

Our simple and inexpensive chromosome preparation protocol can be readily adapted to new invertebrate research organisms to accelerate the discovery of novel genomic patterns across the branches of the tree of life.

## Background

Chromosome preparation is critical to our understanding of animal genetics and genomics. As the price for genome sequencing has dropped, we have witnessed in the last decade an explosive number of research organisms being sequenced and studied. Identifying the basic chromosome composition of an organism sets a foundation to query its unique biological attributes [1, 2]. Behaviors of cells and chromosomes can be fundamentally different in animals that are stable diploids or stable polyploids or those that possess mixed ploidy. Hence, experimental observations at genetic/genomic, tissue, and organismal levels need to be interpreted accordingly.

Isolated chromosomes can be used directly to study multiple questions in genetics and epigenetics. For example, they can be used to collapse scaffolds into chromosomal-level genome assembly [3], to detect insertion or deletion of large fragments of DNA [4], to visualize physical positions of multiple genes [5, 6], to localize histone modifications or to identify DNA regions interacting with chromatin regulators [7, 8]. Moreover, genomes assembled into chromosomes can be used for comparative mapping to study chromosome and genome evolution [9–12].

For communities interested in evolutionary genetics and evolutionary developmental biology, it is favorable to learn about chromosome compositions of multiple species of interest. In these situations, the main challenges come when classical protocols do not work on new research organisms. The development of freshwater planarians as a popular invertebrate for studying regeneration and adult stem cells illustrates many of these challenges and opportunities [13–17]. The existing literature since the early twentieth century on chromosome compositions of freshwater planarians showcased the dynamic changes of ploidy within a species and the wide range of chromosome numbers across closely related species [18]. While these phenomena provided opportunities to study genetics and evolution [19], they also demonstrated the necessity of a standardized chromosome preparation protocol to identify animal ploidy [14].

An optimized chromosomal preparation protocol that can be easily adapted to multiple species would be advantageous. Conventional karyotyping protocols in mammalian cells involve tissue disassociation and cell culture [20], techniques which are not applicable to most invertebrate organisms. Furthermore, solution osmolarity, incubation temperature and time, and centrifugation speed are all complex factors to optimize for karyotyping an invertebrate species of interest. The variety of protocols used in the past for chromosome preparation in planarians and the difficulties in comparing the obtained results [21] clearly demonstrate the necessity to develop a universal protocol that can be easily adapted to as many species as possible.

Here, we optimized a chromosome preparation protocol for chromosome visualization and manipulation in freshwater planarians (phylum Platyhelminthes) and two other research organisms, *Pomacea canaliculata* (phylum Mollusca) and *Nematostella vectensis* (phylum Cnidaria). We demonstrate that (1) both adult and embryonic tissues, treated with colchicine, can provide an adequate number of mitotic cells, (2) deionized (DI) water is a convenient hypotonic reagent, and (3) there is no need for tissue dissociation, centrifugation, or cell culture. We further demonstrate that the chromosomes prepared with this protocol are suitable for downstream applications, including fluorescent in situ hybridization (FISH) in all the tested species. Our protocol is simple and inexpensive, and it can be potentially adapted to a wide variety of invertebrate organisms.

## Methods

### Planarians

Both sexual and asexual strains of *Schmidtea mediterranea*, in addition to asexual strains of *Dugesia japonica*, *Phagocata velata*, and wild planarians collected in the field (Sardinia, Italy) were maintained in the dark at 21–22 °C in 1× Montjuïc water (1.6 mM NaCl, 1.0 mM CaCl_2_, 1.0 mM MgSO_4_, 0.1 mM MgCl_2_, 0.1 mM KCl, and 1.2 mM NaHCO_3_ in DI H_2_O, pH 6.9–8.1) [22]. The above-mentioned species were historically defined by their karyotype, anatomy, and physiological attributes (*e.g.,* asexual reproduction by fission). All animals were fed organic beef liver paste twice per week, and the culture water was exchanged after each feeding. The animals were starved for 1 week before the experiments.

#### Pomacea canaliculata

Specimens of the apple snail *P. canaliculata* were initially obtained from Prof. Davide Malagoli from a population stably maintained at the University of Modena and Reggio Emilia (Italy) [23]. The species was defined based on both morphological features and transcriptome and genome sequencing. *P. canaliculata* specimens were maintained in the lab at 26–27 °C with a 14:10 h light:dark cycle. The animals were housed in tanks filled with artificial freshwater (2.7 mM CaCl_2_, 0.8 mM MgSO_4_, 1.8 mM NaHCO_3_, 1:5000 Remineraliz Balanced Minerals in Liquid Form [Brightwell Aquatics, Fort Payne, AL, USA]). The water was changed twice per week, followed by cleansing of the tanks. The snails were fed ad libitum with lettuce leaves and kale. Deposited egg clutches were collected daily and stored in dry conditions.

#### Nematostella vectensis

Specimens of starlet sea anemone *N. vectensis* Stephenson were initially obtained from Prof. Mark Martindale and Prof. Craig Magie (University of Hawaii, USA). This particular strain was originally collected from the Rhode River (Maryland, USA) and kindly distributed by Dr. Cadet Hand and Prof. Kevin Uhlinger [24, 25]. Species identification was performed based on both morphological features, such as the presence of nematosomes in the gastric cavity, as well as transcriptome and genome sequencing. Colonies of the sea anemone *N. vectensis* were maintained at 18–20 °C in a 1:3 dilution of artificial seawater (12 parts per thousand [ppt] of Instant Ocean Sea Salt) and were fed the larvae of the brine shrimp *Artemia salina* two to five times per week. Spawning induction of sexually mature individuals, egg de-jellying treatment, and fertilization were carried out as previously described [26].

### Planarian sample preparation for chromosome spreads

The posterior or tail region of planarians (~ 2.5 mm) was amputated using a blade, and the regenerating tail fragments were incubated in 0.25% colchicine (Sigma Aldrich) in 1× Montjuïc water for 0 or 6 h in the dark. To test the efficiency of the colchicine treatment, some tail fragments were incubated for 0 or 6 h in 1× Montjuïc water without colchicine.

### *P. canaliculata* sample preparation for chromosome spreads

*P. canaliculata* embryos 5, 6, 8, or 10 days post-fertilization (dpf) were collected, and both the external egg shell and the bright pink perivitelline fluid were removed with forceps. The embryos were incubated in 0.25% colchicine in *Pc*-embryonic salt solution (33.3 mM NaCl, 6 mM KCl, 6.7 mM CaCl_2_, 3.3 mM MgCl_2_, 1.67 mM hydroxyethyl piperazineethanesulfonic acid [HEPES]) for 6 or 24 h in the dark. To test the efficiency of colchicine treatment, some embryos were incubated in *Pc*-embryonic salt solution without colchicine.

Adult snails (2.8–3.2 cm shell length) were incubated in 0.25% colchicine in artificial freshwater for 6, 24, 48, or 72 h. After the incubation, small fragments of the organs of interest (gonads, gills, gut, anterior and posterior kidney, and digestive gland) were dissected with scissors.

### *N. vectensis* sample preparation for chromosome spreads

Fertilized *N. vectensis* embryos were cultured for approximately 5 h post-fertilization (hpf). At the 64- or 128-cell stage, around 200 embryos were transferred to a sterile 50-mm petri dish. They were rinsed twice with 12 ppt artificial seawater to remove extra sperm and incubated in freshly made 0.04% colchicine in 12 ppt artificial seawater for 45 min with gentle rotation in the dark.

### Preparation of siliconized coverslips

Siliconized coverslips (22 × 22 mm) were submerged in Sigmacote® (Sigma Aldrich) for 2–3 s in a fume hood. The coverslips were then propped up vertically for 10 min to dry, rinsed in DI H_2_O for 2–3 s, and propped up vertically to dry.

### Chromosome spread preparation for planarians

Tail fragments were placed in a petri dish after colchicine treatment and rinsed with DI H_2_O. The tissue was punctured using forceps or needles to increase its permeability and then incubated in DI H_2_O for 20 min at room temperature (RT). The samples were fixed with freshly made Carnoy’s fixative (3:1 dilution of methanol:acetic acid) for 30 min on ice. A small portion of the tail fragment was then placed onto a slide using a pair of forceps or a pipette. The sample was soaked in a drop of 60% acetic acid (10–20 μl) and incubated for 5 min. A siliconized coverslip was placed on top of the tissue. The tissue was then squashed by applying constant pressure to the coverslip for 2–3 s to create a single layer of nuclei. Care was taken to keep the coverslip from shifting laterally during its application. After an overnight (ON) incubation at 4 °C, the slides were chilled on dry ice for 10–20 min. While the slides were still sitting on the dry ice, the coverslips were quickly removed using a blade. The blade was inserted between the slide and one corner of the coverslip and used as a lever for removing the coverslip, with care taken not to scratch the slide where the sample was sitting. The slides were subsequently returned to RT, rinsed 3 times with 1× phosphate-buffered saline (PBS), and stained with a 1:5000 dilution of 4′,6-diamidino-2-phenylindole (DAPI) in 1× PBS for 10 min at RT. The slides were then rinsed twice with 1× PBS for 5 min each and mounted with either Prolong Diamond (Molecular Probes) or Vectashield (Vector Laboratories) mounting media. The slides were stored at 4 °C.

### Chromosome spread preparation for *P. canaliculata*

All procedures were the same as those described for the planarian samples except for the following modifications:

- The adult tissues were punctured using a needle, but not the embryos.
- Before tissues were placed on the slides, the older embryos (10 dpf) were cut in half and only the anterior portion was squashed.

### Chromosome spread preparation for *N. vectensis*

All procedures were the same as those described for the planarian samples except for the following modifications:

- Because *Nematostella* embryos are extremely fragile during early cleavage stages, significant caution was taken when changing solutions. Specifically, solutions were added slowly dropwise and enough solution was left in the petri dish to keep the embryos fully submerged to avoid blastomeres being burst as a result of the surface tension exerted by the solutions.
- The embryos were not punctured with a needle and the initial DI H_2_O incubation was performed for only 5 min due to the fragility of the blastomeres.
- After 5 min in 60% acetic acid, the weight of the siliconized coverslip was sufficient to flatten the cells, and no additional pressure was applied.

### Image acquisition and processing

The spreads were imaged on a Zeiss LSM 780 or Zeiss LSM 700 confocal microscope using a 63× or 100× magnification lens. The acquired images were processed and karyograms were generated using Photoshop and Fiji softwares.

### Protocol optimizations

To adapt this protocol for use in other research organisms, the following key variables should be considered:

- If the organism has regenerative potential and adult tissues are to be used, the temporal kinetics of the mitotic peak(s) following amputation may differ from that for the planarians*.*
- Head/trunk fragments with mitotic cells and/or embryos can be used for planarian species with different regenerative capacities.
- Puncturing the tissue with needles to break the external epithelium aids the penetration of DI H_2_O into the tissues and cells. The duration of DI H_2_O incubation, the size of the tissue fragment, the number of perforations made in the tissue, and the colchicine concentration and incubation time are all critical steps to optimize.
- Colchicine treatment is usually recommended since it both increases the yield of metaphase chromosomes and produces more reliable chromosome morphologies [27].
- The pressure applied to the coverslip during the squash is correlated to both size and texture of the tissue fragment. If the applied pressure is too great, the chromosomes may not remain grouped together. However, if the pressure is too minimal, nuclei or chromosomes may overlap.
- Imaging with a confocal microscope is not required, and a common compound epifluorescence microscope could be used as well.
- By varying the length of the colchicine treatment, the level of chromosome condensation can be regulated.
- Chromosome spreads can be used immediately for DNA staining or within a few days for in situ hybridization as reported here. Otherwise, they can be aged for a longer time (if required) for different downstream applications, such as G-banding [28].

### Immunohistochemical staining on planarians

The tail fragments were incubated in 5% *N*-acetyl cysteine (NAC) (Sigma Aldrich) in 1× PBS for 10 min at RT to remove mucous from the epithelium. The tissues were then fixed in pre-chilled Carnoy’s fixative solution (6:3:1 dilution of methanol:chloroform:glacial acetic acid) for 2 h at 4 °C. The samples were then rinsed in 100% methanol. The rehydrated tissues were bleached in 3% formamide and 6% H_2_O_2_ in 0.5% Triton X-100 in 1× PBS for 1 h under direct light. The blocking step in 5% horse serum in 0.3% Triton X-100 in 1× PBS for 1–2 h at RT was followed by the incubation ON in 1:500 Rabbit anti-phospho(Ser10)-Histone H3 (H3P)Ab (Cat. ab-32107, Batch GR37459-28, RRID AB_732930; Abcam) at RT. The samples were rinsed and then incubated in 1:500 Goat anti-Rabbit IgG (H + L) Ab conjugated with Alexa Fluor® 488 (Cat. ab-150081, Batch GR297619-1, RRID N/A; Abcam) ON at RT. The samples were rinsed and mounted with ScaleA2 (4 M urea, 0.1% Triton X-100, 20% glycerol, 2.5% 1,4-diazabicyclo-[2,2,2]-octane [DABCO] in double-distilled water [ddH_2_O]) mounting media.

Z-stack images were acquired with a Nikon Eclipse Ti microscope equipped with a Yokogawa W1 spinning disk head and robotic plate loader. Slides were loaded automatically and imaged with a low magnification objective. Objects were identified using custom software and imaged again with a Plan Apo 20× 0.75 NA air objective. Images were batch stitched and processed using custom plugins and macros in Fiji similar to the procedure in previous reports [29].

### FISH protocol

Chromosome spreads were prepared according to the species-specific protocols described previously through the coverslip removal step. Once the slides were warmed to RT, they were rinsed 3 times with 1× PBS for 5 min each. The slides were then dehydrated in an ice-cold ethanol series (70%, 80%, and 100% ethanol) for 2 min each. Slides were then stored at RT for at least 2 days and as long as 1 month.

The telomere DNA probe (sequence [TTAGGG]×7) was obtained from Integrated DNA Technologies (USA) and labeled with digoxigenin (DIG)-deoxyuridine triphosphate (dUTP) using the recombinant Terminal Transferase (Roche) according to the manufacturer’s protocol. For each slide, 20 ng of labeled telomere DNA probe was mixed with 10 volumes of Master Hybridization Mix (4× saline-sodium citrate buffer [SSC], 20% dextran sulfate, 2 mg/ml nuclease-free bovine serum albumin [BSA], 50% deionized formamide in ddH_2_O). The total volume (~ 22 μl) was placed on the area containing the chromosome spreads, covered with a 22 × 22 mm coverslip, and sealed with mineral oil. The slides were incubated for 5 min at 70 °C to denature the DNA, followed by 24–36 h at RT for hybridization. Next, the slides were submerged in 2× SSC in a slide staining jar to remove the coverslips and then rinsed successively with 2× SSC, 0.5× SSC, and TNT (100 mM Tris-HCl, 150 mM NaCl, 0.1% Tween 20) for 15 min each at RT. The areas containing the chromosome spreads were covered with 1:200 anti-DIGAb conjugated with Rhodamine (Cat. 11207750910, Batch N/A, RRID AB_514501; Roche) and 1:1000 DAPI in TNB (5% fetal bovine serum [FBS] in TNT) in the dark for either 1–4 h at RT or ON at 4 °C. The slides were rinsed with TNT for 15 min at RT and then mounted, imaged, and stored at 4 °C.

### Statistical analysis

An unpaired two-sample *t* test was performed for calculation of the statistical difference between the number of H3P-positive cells in tail fragments at 0 h post-amputation (hpa) incubated with (*N* = 20) and without colchicine (*N* = 19) and at 6 hpa incubated with (*N* = 18) and without colchicine (*N* = 17). The differences were considered statistically significant with *P* < 0.05.

## Results

### Optimization of a chromosome preparation protocol

**Fig. 1:**
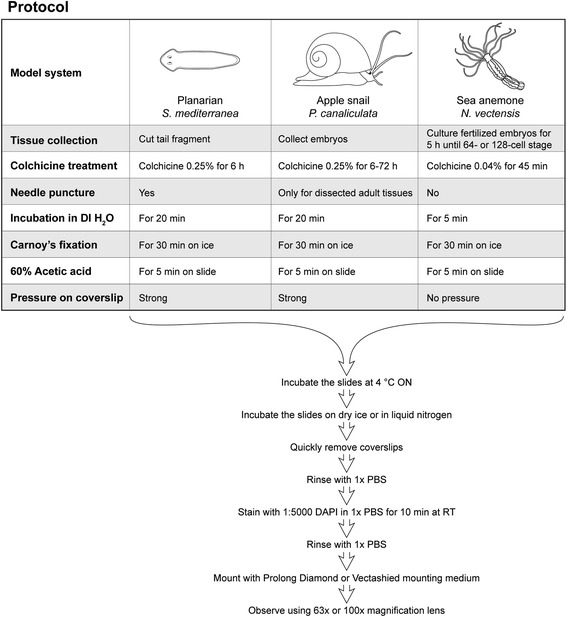
Schematic illustrating the chromosome preparation protocols optimized for each model organism. Key steps in the chromosomal spread protocol are summarized as a flowchart for three invertebrate species. The first part of the protocol consists of steps that require species- and tissue-specific optimization. Each column highlights similarities and differences among the procedures for the planarian *S. mediterranea*, the freshwater snail *P. canaliculata*, and the sea anemone *N. vectensis*. The second part of the protocol did not require species-specific optimization and was standardized for all organisms tested

Adult tissues containing mitotic cells or entire embryos were collected and processed for karyotype analysis (Fig. 1).

**Fig. 2:**
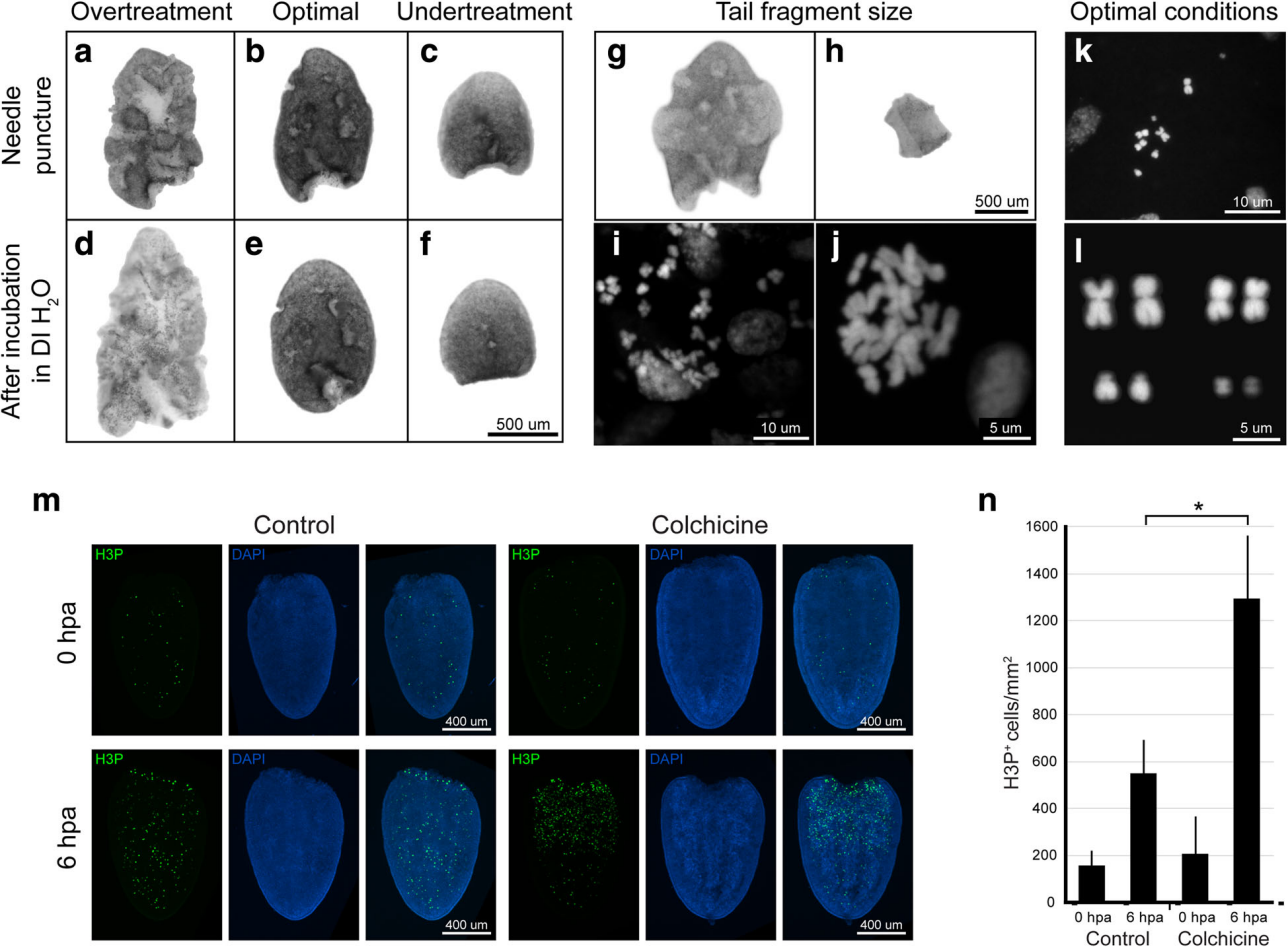
Protocol optimization in the freshwater planarian *S. mediterranea.* Conditions critical for optimal chromosome preparation in planarians include extent of needle puncture and colchicine treatment. An excessive number of needle punctures can cause deformation and disaggregation of the tissue, which became too swelled and fragile after incubation in DI H_2_O (**a, d**). This led to dispersed chromosomes on the slide without distinguishable cell boundaries (**i**). An optimal number of needle punctures (4~10 in a 0.5 × 1 mm fragment) facilitated tissue swelling without causing disaggregation in DI H_2_O (**b, e**) and maintained tissue integrity after acetic acid treatment (**g**). This produced optimal spreading of chromosomes on a slide (**k, l**). An insufficient number of needle punctures did not swell the tissues in DI H_2_O (**c, f**), which led to overlapping chromosomes (**j**). One fourth of a 0.5 × 1 mm fragment (**g, h**) can produce sufficient optimal chromosome spreads. If too large a fragment is used for squashing, it will also result in crowded chromosomes (**j**). Colchicine treatment increased the number of mitotic cells by ~ 2.5 times (**m, n**, *P < 0.0001). Mitotic cells were labeled with anti-H3P Ab (**m**)

For freshwater planarians, tail fragments were used for karyotyping (Fig. 2). In our experience, puncturing tail fragments with a needle while they are immersed in DI H_2_O was essential to obtain optimal and robust results, because punctures likely facilitate the penetration of DI H_2_O into tissues. With more than 10 punctures in a 0.5 × 1 mm-sized tail fragment, the wounds did not heal properly, which led to excessive exposure of the internal tissues to DI H_2_O (Fig. 2a, d). These tissues fell apart during the 60% acetic acid treatment and yielded only a few intact chromosome spreads because the majority of chromosomes were scattered without discernable cell boundaries (Fig. 2i). With few to no injuries, the tissues and cells of the tail fragment did not swell enough during DI H_2_O treatment (Fig. 2c, f). Spreads from these tissues tended to have very crowded chromosomes (Fig. 2j) or chromosomes that overlapped with interphase nuclei, making an accurate analysis of chromosome numbers and morphologies difficult. A properly punctured tail fragment increased slightly in size after DI H_2_O treatment (Fig. 2b, e). It expanded more, but retained integrity after 60% acetic acid treatment (Fig. 2g). A tail fragment could be sliced into several smaller pieces (250 × 250 μm) (Fig. 2h), and one of those was sufficient to produce enough chromosome spreads with optimal morphologies. If the tissue used for the squash was too big, the chromosome spreads produced could be crowded and overlapping (Fig. 2j). With an optimal number of punctures, tissue swelling, tissue size, and pressure applied on the coverslip, an optimal karyotype could be obtained (Fig. 2k, l), where the chromosomes did not overlap or mix with chromosomes from other nuclei.

Treatment with the mitosis-inhibiting drug colchicine significantly increased the number of mitotic cells in planarians (Fig. 2m, n), similarly as in other systems [30]. When colchicine binds tubulin, it prevents spindle formation and halts dividing cells in metaphase. An antibody detecting phospho(Ser10)-Histone H3 (H3P) was used to assess the number of mitotic cells in a planarian tail with or without 6 h of colchicine treatment. In the absence of colchicine, the number of dividing cells approximately tripled at 6 hpa compared to baseline (0 hpa). Hence, with colchicine treatment, the number of mitotic cells increased about seven times by 6 hpa compared to baseline, yielding a significantly higher number of chromosome spreads in the tissue (Fig. 2n).

### Applicability of the protocol to multiple planarian species

**Fig. 3:**
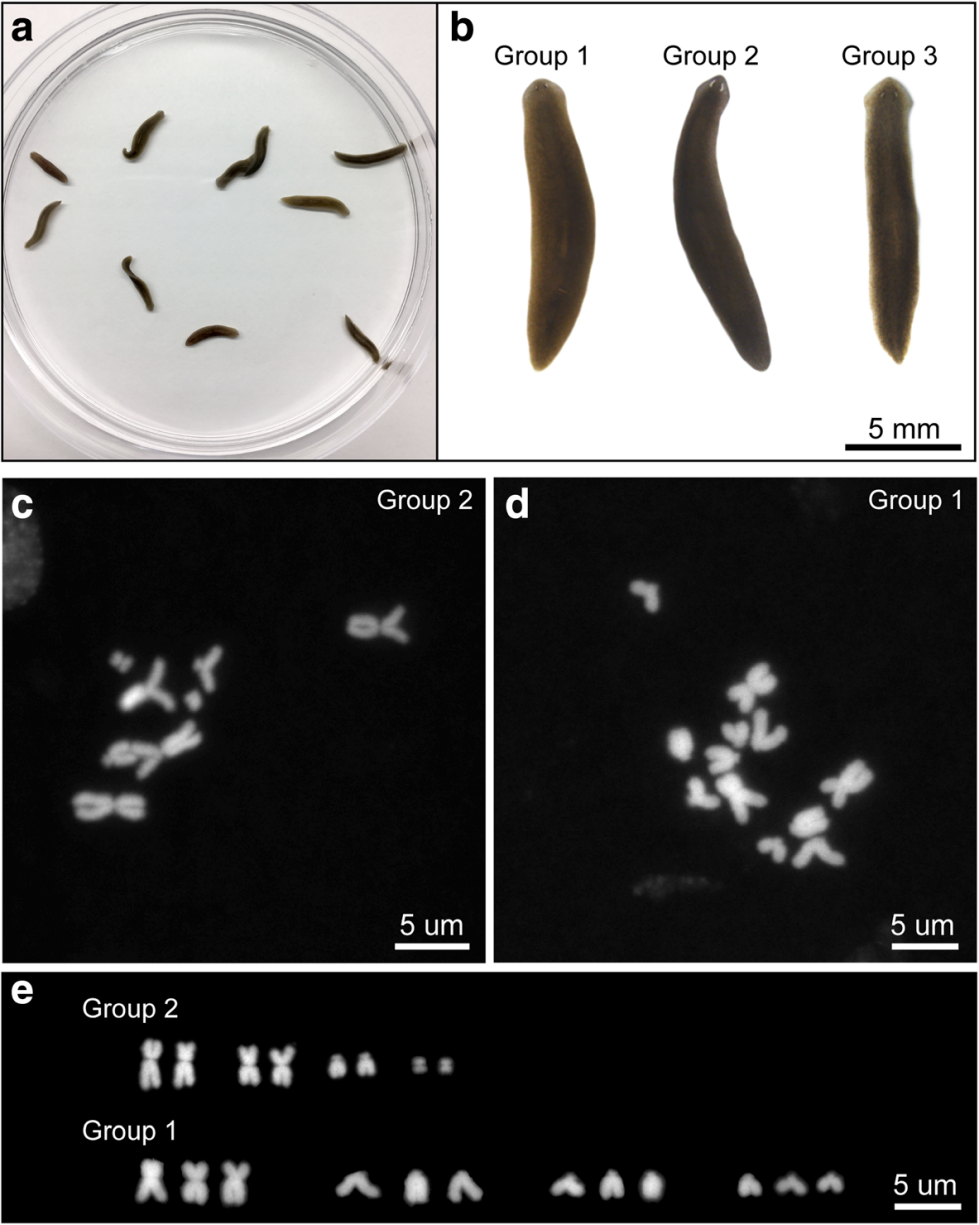
Species identification through karyotyping of wild planarians. Clearly distinct karyograms were obtained from three groups of morphologically similar planarians obtained in the wild*.* **a** A mixed population of planarians collected in Sardinia (Italy). **b** Wild planarians grouped based upon their gross morphology. **c** Karyotype belonging to planarians in the second morphological group. This karyotype is the same as the *S. mediterranea* karyotype*.* **d** Karyotype for planarians belonging to the first morphological group, which is *Schmidtea polychroa.* **e** Karyogram of the planarians belonging to the second and first morphological groups, which are diploid and triploid organisms, respectively

To survey genome heterozygosity and diversity in populations of *S. mediterranea*, wild planarians were collected in the field in Sardinia (Italy) [19] (Fig. 3a). Under a dissecting microscope, we defined three different morphological groups based on the shapes of the head and eyes and the body pigmentation (Fig. 3b). These features, however, were not sufficient to discern distinct species. We used the chromosome preparation protocol optimized on *S. mediterranea* to karyotype the planarian species present in this wild-caught population. Planarians in the second morphological group were diploid with 2n = 8 chromosomes (Fig. 3c, e), while those in the first morphological group were triploid with 3n = 12 chromosomes (Fig. 3d, e). Hence, we were able to readily determine that animals belonging to the second morphological group were *S. mediterranea* because they had the same karyotype as previously documented (Fig. 2k, l) [31].

**Fig. 4:**
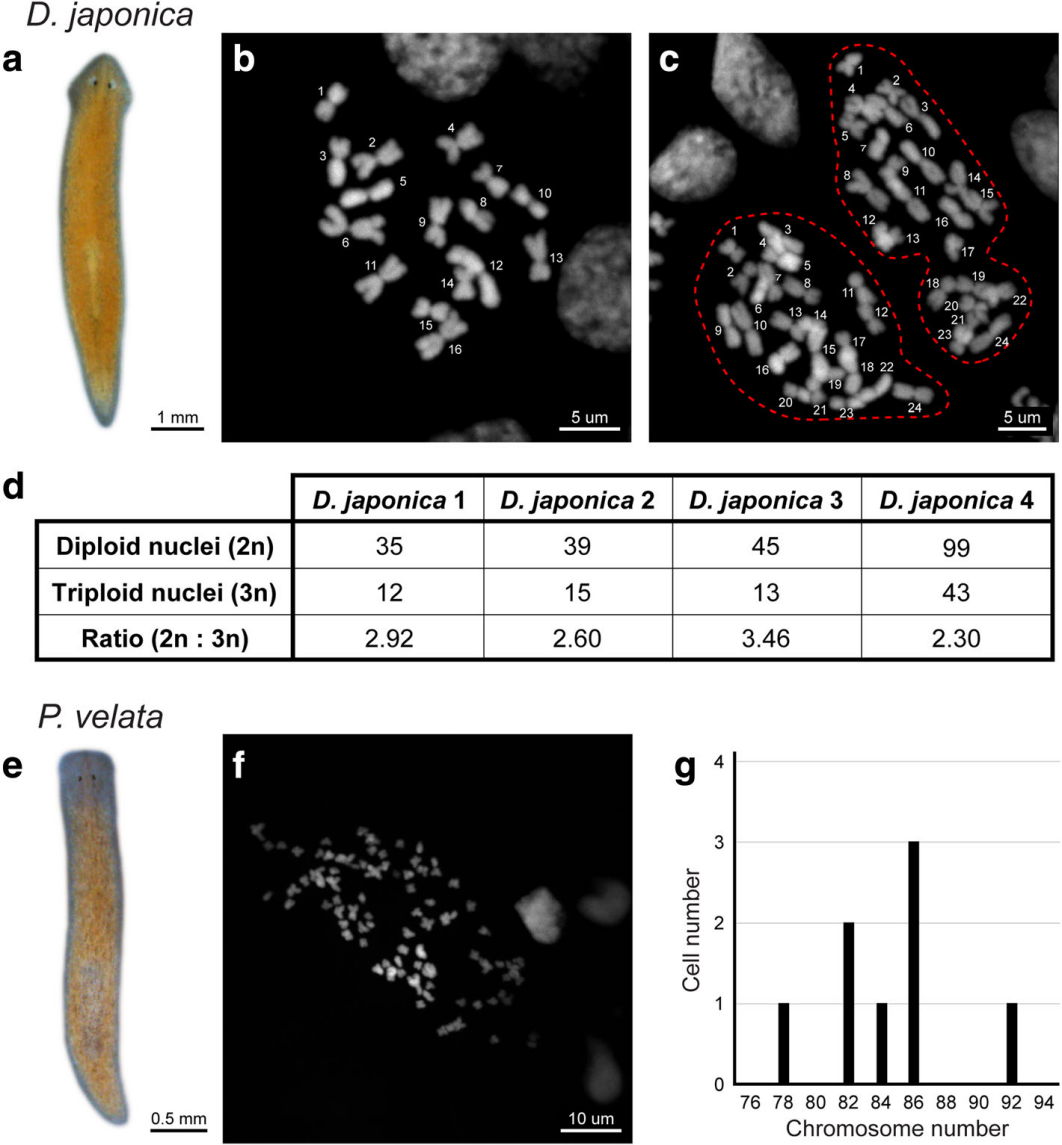
The optimized chromosome preparation protocol allows karyotyping of multiple planarian species. The optimized chromosome preparation protocol was used to generate karyotypes for two additional planarian species. **a** *D. japonica.* **b, c** Karyotypes obtained for *D. japonica* demonstrating the presence of both diploid (**b**) and triploid (**c**) cells in the same individual. **d** Table summarizing the ratio of diploid to triploid dividing cells in four *D. japonica* individuals*.* **e** *P. velata.* **f, g** A representative *P. velata* karyotype (**f**) and quantification of the number of chromosomes per cell (**g**)

This optimized and simple karyotyping protocol also works in planarian species other than *S. mediterranea.* First, we applied the protocol to *Dugesia japonica*, a planarian species reported to have mixed ploidy in different cells of the same animal [32] (Fig. 4a). Indeed, during our analysis, multiple individuals were identified with mitotic cells that were either diploid (2n = 16) (Fig. 4b) or triploid (3n = 24) (Fig. 4c). The co-existence of diploid and triploid adult dividing cells in the same animal was intriguing. We quantified the ratio of diploid to triploid mitotic cells in *D. japonica.* Interestingly, there were consistently more diploid cells than triploid cells in all four animals examined, and the ratio varied from 2.30 to 3.46 with an average of 2.8 (Fig. 4d).

Second, we applied the protocol to *Phagocata velata*, a fissiparous species that reproduces asexually by fission [33] (Fig. 4e). The protocol worked successfully on its tail fragments, and we observed that this species has the largest number of chromosomes in planarians reported thus far (Fig. 4f). The specific number of chromosomes seems to vary from cell to cell, but on average there are about 84 chromosomes per nucleus (Fig. 4g). Altogether, the method reported here was successful in rapidly generating unambiguous karyotypes for a diverse cohort of planarian species.

### The protocol is broadly applicable to species belonging to different phyla

**Fig. 5:**
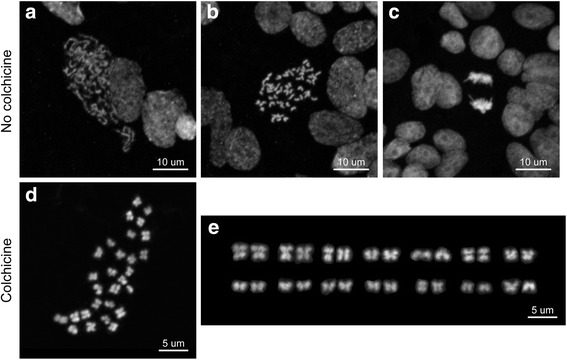
Chromosome preparation in the freshwater snail *P. canaliculata.* The optimized chromosome preparation protocol in planarians was used to generate karyotypes for both adult and embryonic tissues of the freshwater snail *P. canaliculata.* **a–c** Mitotic cells showing **(a)** early prophase, **(b)** late prophase or metaphase, and **(c)** anaphase in embryos 5 dpf without colchicine treatment. **d** Karyotype and **e** karyogram obtained from *P. canaliculata* embryos 5 dpf treated with colchicine. They are also representative of chromosome spreads obtained from the anterior and posterior kidney and the digestive gland in adult snails

The planarian optimized protocol was successfully applied to the apple snail *P. canaliculata* with minimal modifications*.* To obtain chromosomes for karyotyping, we first applied this method on embryos 5 dpf, which have tissues undergoing abundant and frequent cell divisions. We found that, in the absence of colchicine treatment, dividing cells in various phases of mitosis were visible, but chromosomal compaction was not uniform (Fig. 5a–c). Optimal chromosome spreads are usually obtained from cells in metaphase with highly condensed chromosomes; therefore, colchicine treatment seems to be required. As in planarians, a treatment with colchicine for 6 h in 5 dpf embryos overcame the problem of insufficient chromosome compaction and yielded many cells blocked in metaphase that had well-condensed chromosomes (Fig. 5d). *P. canaliculata* nuclei were diploid and the number of chromosomes was consistent (2n = 28 chromosomes). Moreover, a simple analysis of the size and shape of the chromosomes through ImageJ software allowed us to organize them in the karyogram (Fig. 5e).

To further test the adaptability of this protocol on different tissues, we used it on various adult organs of *P. canaliculata*. After an incubation for 72 h in colchicine, the snails were dissected to collect organs known to undergo rapid cell turnover including gonads, gills, gut, anterior and posterior kidney, and digestive gland. We obtained good chromosome spreads from the anterior and posterior kidney and the digestive gland (representative karyotype and karyogram are shown in Fig. 5d, e). The tissue producing the most chromosome spreads was the gonads, but they contain many dividing polyploid cells (unpublished data), which could result in a misleading karyotype analysis. Shorter colchicine incubation (6, 24, and 48 h) provided no or extremely rare mitotic cells in the analyzed adult tissues.

**Fig. 6:**
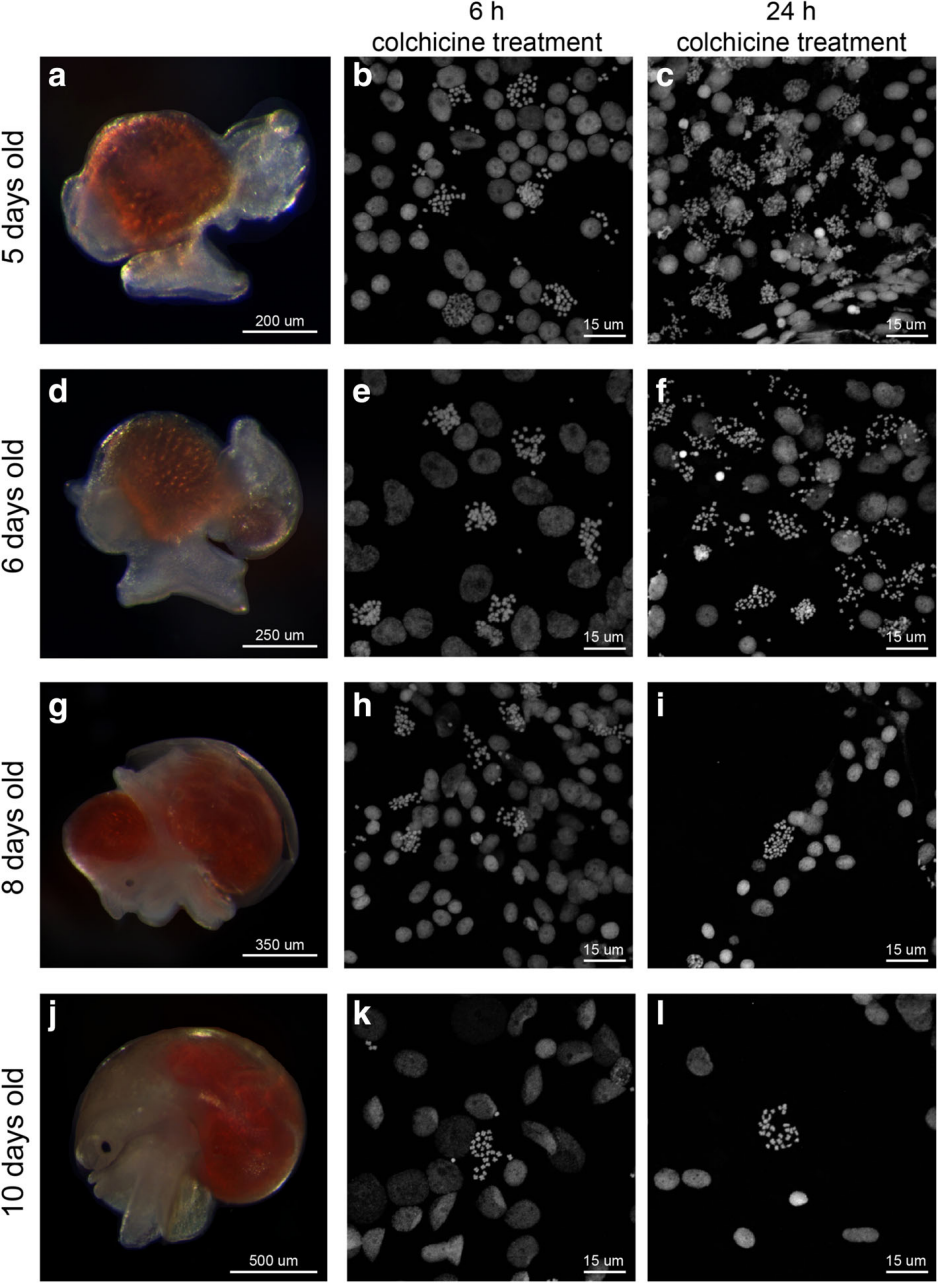
Chromosome spreads for different embryonic stages of *P. canaliculata*. The duration of colchicine treatment on embryos resulted in different densities of mitotic chromosomes for different embryonic stages. **a, d, g, j** *P. canaliculata* embryos 5, 6, 8, and 10 dpf, respectively, after removal of egg shell and perivitelline fluid. **b, e, h, k** Chromosome spreads obtained from embryos at indicated developmental stages incubated in colchicine for 6 h. **c, f, i, l** Chromosome spreads obtained from embryos at indicated developmental stages incubated in colchicine for 24 h

Since the protocol successfully worked on both 5 dpf embryos and adult tissues of *Pomacea* by varying the time of colchicine treatment*,* we tested if this protocol is adaptable to other embryonic stages. We showed that a good karyotype can be obtained from any source of tissues by simply adjusting the time of the colchicine incubation (Fig. 6). Embryos 5, 6, 8, and 10 dpf (Fig. 6a, d, g, j) were treated with colchicine for 6 h and used for chromosome preparation. We observed that younger embryos provided more spreads, but the density of dividing cells was too high in certain areas of the slide, making it difficult to distinguish cell boundaries (Fig. 6b). The older embryos, on the other hand, had a lower density of mitotic cells (Fig. 6e, h, k). We concluded that embryos 6 to 10 dpf treated with colchicine for 6 h produced enough chromosome spreads that were sufficiently separated from one another to easily associate them with individual cells. A longer incubation in colchicine was also tested. After 24-h colchicine treatment, the chromosomes of 5 and 6 dpf embryos were found to be crowded and cell boundaries were hard to define. At these stages, 24-h colchicine treatment would be useful only if large numbers of chromosomes are needed, such as for chromosome sorting (Fig. 6c, f). For older embryos (8 and 10 dpf), 24-h colchicine treatment resulted to be optimal for karyotype analysis (Fig. 6i, l). Hence, a general rule of thumb in chromosome preparation in snails is that the older the tissue, the longer the colchicine treatment must be.

**Fig. 7:**
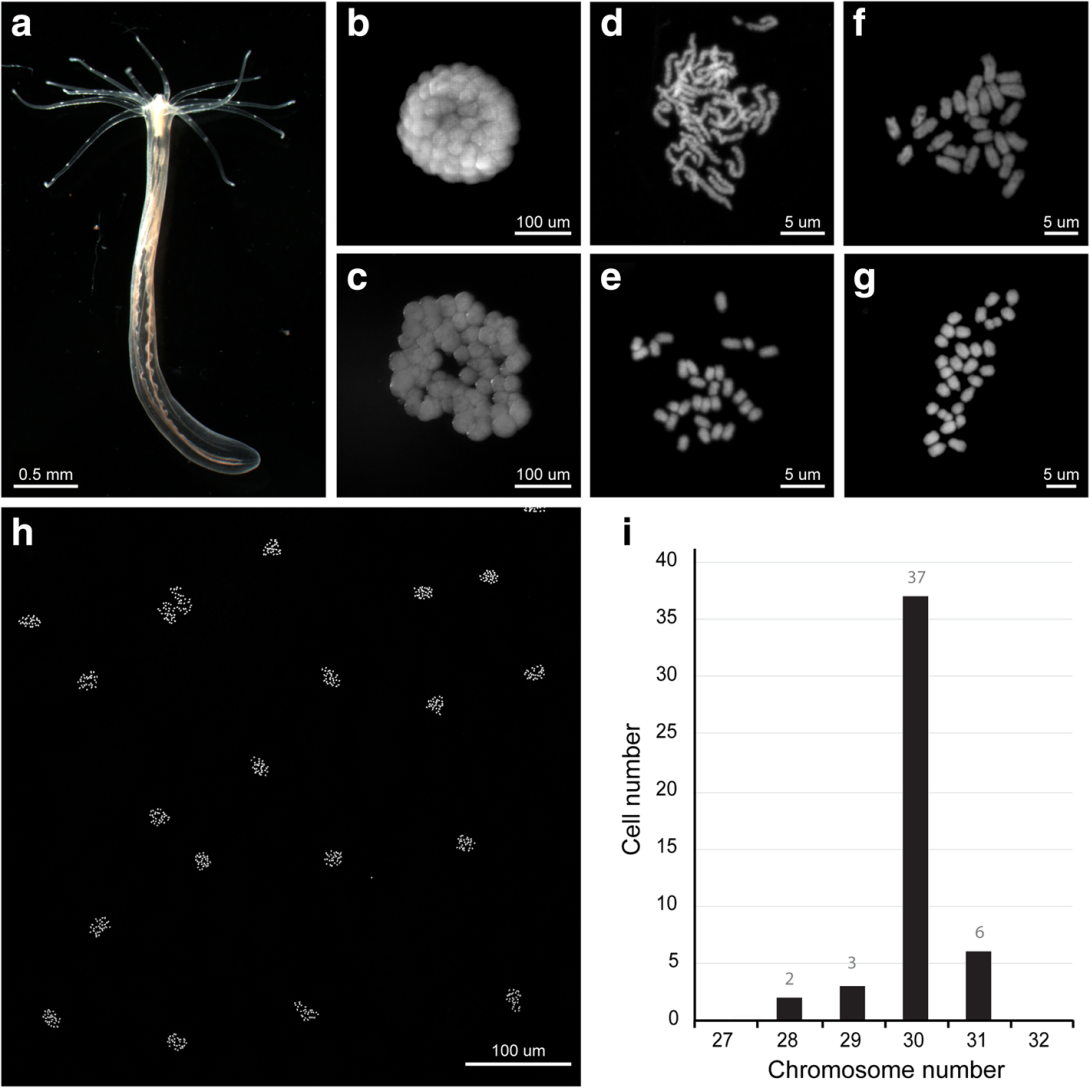
Chromosome preparation in the embryos of the sea anemone *N. vectensis.* The optimized chromosome preparation protocol was used to generate karyotypes for embryos of the sea anemone *N. vectensis.* **a** Juvenile stage of *N. vectensis*. **b, c** A 128-cell embryo (**b**) before and (**c**) after 5 min DI H_2_O incubation. **d** Karyotype obtained from *N. vectensis* embryos after a 30-min incubation in 0.04% colchicine. Notice the incomplete condensation of chromosomes. **e** Karyotype obtained from *N. vectensis* embryos after a 45-min incubation in 0.04% colchicine. (2n = 30). **f**, **g** Excessive DI H_2_O incubation or physical force during preparation results in alteration of chromosome morphology and chromosome loss (**f** 2n = 25, **g** 2n = 29). **h** Chromosome spreads obtained using 128-cell embryos imaged under lower magnifications. All blastomeres were synchronously arrested at metaphase. **i** Quantification of the number of chromosomes per cell

Next, we tested the chromosome preparation protocol in a phylogenetically more distant species, the cnidarian *N. vectensis* (Fig. 7a). Embryos between the 64- and 128-cell stage were chosen as sources of mitotic cells (Fig. 7b, c). At this stage, cell divisions are still synchronous with a 45-min interval. We showed that the best chromosome spreads were obtained with a 45-min treatment with 0.04% colchicine (Fig. 7d, e). Nearly all blastomeres were arrested in metaphase and displayed well-condensed chromosomes. A 5-min osmolarity shock in DI H_2_O and a quick fixation using Carnoy’s fixative solution provided sufficient separation between individual chromosomes. Up to 10 fixed embryos were placed on one glass slide with a coverslip gently placed on top. The weight of the coverslip naturally flattened the embryos, achieving minimal overlapping between neighboring cells (Fig. 7h). When the DI H_2_O treatment was too long or when additional pressure was applied to the coverslip, we observed frequent chromosome loss, as well as alterations in chromosome morphology in the final spread (Fig. 7f, g). In contrast to other species examined in this study, *N. vectensis* sister chromatids tended to remain in close proximity to each other, making the chromosomes appear “bar-shaped” instead of the more classic “X” shape (Fig. 7d, e). Subsequent quantification of chromosome numbers in 48 blastomeres confirmed the observation reported by Putnam et al. [34], who showed that *N. vectensis* nuclei are diploid (2n = 30) (Fig. 7i).

### Chromosome preparation protocol is compatible with downstream applications

**Fig. 8:**
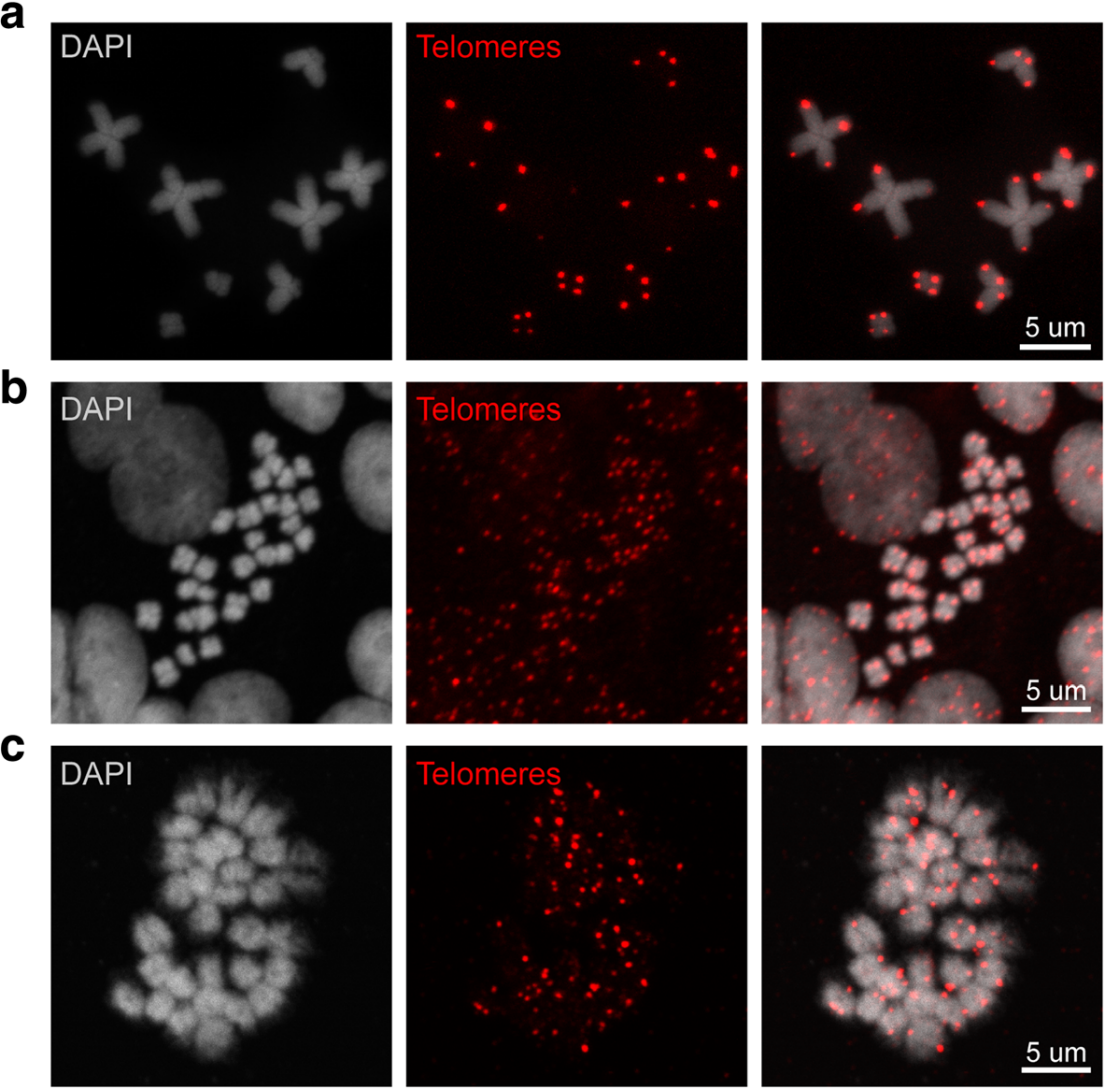
Telomere FISH on chromosome spreads of three model organisms. The chromosome preparation protocols optimized for each species are compatible with chromosomal FISH. Chromosomes spreads obtained from **a** *S. mediterranea*, **b** *P. canaliculata,* and **c** *N. vectensis* were successfully stained with telomere-specific DNA probes labeled with DIG-dUTP

To test whether our protocol can provide chromosomes suitable for further downstream analyses other than the assembly of karyograms, we used highly conserved telomere-specific DNA probes labeled with DIG-dUTP (sequence [TTAGGG]_*n*_) [35] for chromosomal FISH. Specific and intense fluorescent signals at the ends of each sister chromatid were observed in all chromosomes obtained from adult *S. mediterranea* (Fig. 8a), *P. canaliculata* embryos 6 dpf (Fig. 8b), and 128-cell stage embryos of *N. vectensis* (Fig. 8c).

## Discussion

Techniques providing easy and inexpensive access to chromosome composition (*i.e.*, number and morphology) are invaluable for our understanding of chromosome behaviors and genome architecture in any organism of interest. Here, we reported an optimized protocol to prepare chromosomes for karyotyping and FISH in species belonging to three different phyla: Platyhelminthes, Mollusca, and Cnidaria. We expect that the protocol reported here will be highly adaptable to the study of multiple invertebrate species.

In planarians, the analysis of karyotypes across multiple species was pioneered by Benazzi and his coworkers [18, 36] and spanned from the early to late twentieth century. This body of work identified a large cohort of sexual and asexual planarian species and provided information on both the karyotypes and anatomy of sexual reproductive systems. However, few detailed karyotyping methodologies are available. Traditional methods employed regenerating blastemas 3 dpa for the preparation of chromosome spreads [37], which provided very little material to work with, as the blastema are usually rather small. Furthermore, the traditional methods required a long processing time. Another issue associated with this literature is that the conditions for chromosome preparation are highly variable from researcher to researcher. Here, we showed that a fragment of tissue amputated from adult worm and treated with colchicine for 6 h and successively with DI water as hypotonic reagent is sufficient to yield well-spread chromosomes in multiple planarian species. This can potentially function as a standardized methodology for planarian chromosome preparation.

Recently, interest in the apple snail *P. canaliculata* (originally from South America) is significantly rising among researchers. This snail is listed among the 100 most invasive species worldwide (Global Invasive Species Database). Nowadays, many studies are focused on both the characterization of its immune system and a search for methods to limit its diffusion [38–40] but also on establishing this animal as a molluscan research organism for laboratory research [41–43]. We confirmed that the *P. canaliculata* genome is diploid with 2n = 28 chromosomes [44, 45], applying the protocol optimized on planarian to both embryos at different developmental stages and adult tissues. It is important to highlight that only a small number of optimizations were required to successfully isolate a significant number of good-quality chromosome spreads.

*Nematostella* has gained attention over the last decade due to both its unique phylogenic position as an early branching metazoan and its amenability to experimental manipulation. The sequencing of the *Nematostella* genome in 2007 provided crucial insights into the ancestral eumetazoan gene repertoire and evolutionarily conserved signaling pathways [34]. In contrast to its apparent morphological simplicity, *Nematostella* possesses a complex genome [46, 47], whose sequence and high-quality annotation have provided a robust platform for applying modern molecular biological tools such as genome editing to study gene function in the context of evolution [48, 49]. Yet, despite the rapid expansion of *Nematostella* as a research organism, little attention has been paid to its chromosomal structure and organization. Previous scattered experimental evidence showed that *Nematostella* is a diploid animal with chromosome number 2n = 30 [34]. Interestingly, this number is identical to those of several other hydrozoans and it is also comparable to those of several species of *Acropora* corals (2n = 28) [50, 51]. Utilizing the karyotyping method discussed above on early stage embryos (64- and 128-cell stage) and optimizing a few steps, we easily obtained highly synchronized metaphase spreads.

Many times, karyograms have been used as a powerful tool for species identification, among both planarians and mollusks [21, 52]. We showed that when planarians are collected in the wild, it can be difficult to identify the species only on the basis of morphological features. The high regenerative potential of these animals allows the researcher to karyotype each individual without sacrificing it. Historically, planarian species identification relies on the anatomy of sexual reproductive organs [18, 53, 54], which leaves the cohort of asexual planarian species largely uncatalogued. A proper characterization of the karyotypes of both sexual and asexual species would facilitate the identification and cataloging of planarian species, while also providing a rich resource for the understanding of karyotype evolution [21]. Similarly, over the years, the Gastropoda class has been reorganized multiple times in subclasses and families, and new species have been integrated, but the lack of a unique and established method for species identification has created multiple incongruences [55, 56]. In particular, Hayes and colleagues faced this problem within the *Ampullariidae* family, suggesting a combination of morphological and molecular features for species identification [57, 58]. The karyogram is potentially a very useful feature that can be added to the plethora of characteristics required for species identification [44, 52, 59, 60]. The possibility to easily obtain unambiguous karyotypes from material collected in the field irrespective of embryonic developmental stages or from adult tissue without extensive optimization stands to be extremely useful in comparative analyses and population genetic studies.

## Conclusions

In conclusion, we optimized a chromosome preparation protocol in invertebrate species belonging to three phyla. The protocol is simple, inexpensive, and highly adaptable to multiple species. Prepared chromosomes can also be used for multiple downstream applications including FISH for localizing telomeres and potentially any other gene of interest.
